# Depression and Heart Failure in US Veterans

**DOI:** 10.1001/jamanetworkopen.2025.9246

**Published:** 2025-05-08

**Authors:** Jamie L. Pfaff, Svetlana K. Eden, Suman Kundu, Charles W. Alcorn, Jonah Garry, Robert A. Greevy, Jesse C. Stewart, Matthew S. Freiberg, Evan L. Brittain

**Affiliations:** 1 Department of Medicine, Vanderbilt University Medical Center, Nashville, Tennessee; 2 Department of Biostatistics, Vanderbilt University Medical Center, Nashville, Tennessee; 3 Division of Cardiovascular Medicine, Department of Medicine, Vanderbilt University Medical Center, Nashville, Tennessee; 4 University of Pittsburgh School of Public Health, Pittsburgh, Pennsylvania; 5 Center for Health Services Research, Vanderbilt University Medical Center, Nashville, Tennessee; 6 Department of Psychology, Indiana University Indianapolis, Indianapolis; 7 Geriatric Research Education and Clinical Centers (GRECC), Veterans Affairs Tennessee Valley Healthcare System, Nashville

## Abstract

**Importance:**

Depression and heart failure (HF) affect millions of US adults. Incident HF risk following depression diagnosis is understudied.

**Objective:**

To examine the association between incident HF and prevalent depression among veterans.

**Design, Setting, and Participants:**

This cohort study analyzed sociodemographic and clinical data of US veterans participating in the Veterans Affairs (VA) Birth Cohort. Data were obtained from the VA Corporate Data Warehouse between January 1, 2000, and October 1, 2015. Participants were born between 1945 and 1965, were free of HF at baseline, and met a medical home definition (had 3 outpatient visits within 5 years). Statistical analysis was performed from May 2022 to February 2025.

**Exposures:**

Prevalent depression, defined as 1 inpatient or 2 outpatient visits with *International Classification of Diseases, Ninth Revision (ICD-9)* or *International Statistical Classification of Diseases and Related Health Problems, Tenth Revision (ICD-10)* codes for depression.

**Main Outcomes and Measures:**

The primary outcome was time to incident HF, defined as time from baseline to documented date of the first inpatient or second outpatient visit with *ICD-9* or *ICD-10* codes for HF. A Cox proportional hazards regression model adjusted for relevant covariates was used to assess the association of depression and incident HF.

**Results:**

A total of 2 843 159 veterans (median [SD] age, 54 [49-59] years; 2 677 919 males [94.2%]; 556 914 [19.6%] self-identified as Black, 144 485 [5.1%] as Hispanic, 1 975 068 [69.5%] as White, and 99 011 [3.5%] as other race and ethnicity) were included. Participants were followed up for incident HF over a median (IQR) duration of 6.9 (3.4-11.0) years. Generally, 8.0% of participants (226 247 of 2 843 159) had prevalent depression at baseline. Females made up a larger percentage of those with vs without depression (11.4% vs 5.3%). Participants with depression demonstrated higher unadjusted incident HF rates compared with those without depression (136.9 [95% CI, 132.2-141.7] cases per 10 000 person-years vs 114.6 [95% CI, 113.4-115.9] cases per 10 000 person-years, respectively). After adjusting for sociodemographic and cardiovascular risk factors, depression was associated with an increase in incident HF hazard of 14.0% (hazard ratio [HR], 1.14; 95% CI, 1.13-1.16), with an estimated adjusted median (IQR) incidence rate difference of 16.0 (14.9-18.3) cases per 10 000 person-years. Among patients without comorbidities, depression was associated with a higher increase in incident HF hazard (HR, 1.58; 95% CI, 1.39-1.80), with an estimated adjusted median (IQR) rate difference of 14.2 (9.5-19.5) cases per 10 000 person-years.

**Conclusions and Relevance:**

In this cohort study, depression among veterans was associated with an increased hazard of incident HF after controlling for demographic and cardiovascular risk factors. Higher incident HF rates in patients with depression remained consistent in an otherwise low-risk cohort.

## Key Points

**Question:**

Are veterans with depression at elevated risk of heart failure?

**Findings:**

In this cohort study of 2 843 159 veterans, depression was associated with a 14% increased hazard of incident heart failure independent of traditional sociodemographic and cardiovascular risk factors.

**Meaning:**

The findings suggest that veterans with comorbid depression have a higher risk for heart failure independent of other risk factors.

## Teaser

This cohort study examines the risk, adjusted for sociodemographic and cardiovascular factors, of developing incident heart failure among US veterans with depression vs without depression.

## Introduction

As the US population ages, the prevalence of heart failure (HF) continues to increase, with approximately 6.7 million adults with HF (according to 2017-2020 data).^1,2^ Among patients with HF, at least 1 in 5 experience depression, a comorbid condition associated with higher rates of hospitalization and mortality.^3,4,5,6^ Less is known about the risk of incident HF following a diagnosis of depression; however, prior findings of increased rates of HF following a diagnosis of depression suggest an association between the 2, and there is evidence that cardiovascular disease prognosis can improve with depression remission.^7,8,9^ In 2021, approximately 21 million US adults experienced at least 1 major depressive episode, increasing in recent years, with the highest rates among those aged 18 to 25 years.^10,11^ The subpopulations at greatest risk for comorbid HF and depression remain underrecognized. Adults without other medical comorbidities, women, veterans, and racial and ethnic minoritiy groups may be especially susceptible due to higher rates of depression or underdiagnosis.^10,11,12,13,14,15^

Our objective was to examine the association between incident HF and prevalent depression among veterans. We used data from the US Department of Veterans Affairs (VA) Birth Cohort, a sample at least 1 magnitude larger than any US study examining these comorbid conditions to date.^16^ We hypothesized that having depression would be associated with a higher hazard of incident HF.

## Methods

### Study Design and Participants

The study population was derived from participants in the VA Birth Cohort, an observational, prospective cohort study of approximately 3.4 million US veterans born between 1945 and 1965 receiving care at Veterans Health Administration (VHA) facilities. The VA Birth Cohort data are fully deidentified and approved for analysis through a waiver of patient informed consent. The VA Tennessee Valley Healthcare System Institutional Review Board approved this cohort study. We adhered to the Strengthening the Reporting of Observational Studies in Epidemiology (STROBE) reporting guideline.^17^

We extracted sociodemographic and clinical data for these participants from the VA Corporate Data Warehouse between January 1, 2000, and October 1, 2015. For this analysis, we included participants who were free of HF at baseline and who met a medical home definition (3 outpatient visits within 5 years). Additionally, participants were excluded due to being younger than 18 years, having unknown sex data, death within 1 year of meeting medical home definition, or receiving a heart transplant prior to or within 1 year of meeting medical home definition.

### Variables and Measurements

#### Outcomes

The primary outcome was time to incident HF. We defined incident HF as the presence of 1 inpatient or 2 outpatient visits with VA, VA fee-for-service, or Medicare *International Classification of Diseases, Ninth Revision (ICD-9)* or *International Statistical Classification of Diseases and Related Health Problems, Tenth Revision (ICD-10)* codes for HF (425.x, 428.x, I42.x, and I50) in the electronic health record. Depression was defined as the presence of 1 inpatient or 2 outpatient visits with VA, VA fee-for-service, or Medicare *ICD-9* or *ICD-10* codes for depression (296.20-26, 296.30-36, F32.0-5, F32.9, F33.0-1, F33.3-4, F33.41-42, and F33.9) in the electronic health record. Medical home was defined at the date of the third outpatient visit occurring within a 5-year span. The baseline date was defined at 1 year after the medical home date. Time to incident HF was defined as the time from baseline to documented date of the first inpatient or second outpatient visit with *ICD-9* or *ICD-10* codes for HF.

#### Covariates

We collected data on covariates closest to the medical home date, allowing for values up to 180 days after the medical home date. Sociodemographic data, including age, sex, and race and ethnicity, were collected from VHA administrative data. Self-identified race and ethnicity categories included Black, Hispanic, White, or other (all other self-identified race groups). We created the other category because several individual race and ethnicity groups were small and underpowered to analyze separately. Clinical inpatient, outpatient, and laboratory data were collected from the Corporate Data Warehouse. We also collected data on the following covariates: depression, diabetes, coronary artery disease (CAD), hypertension, ischemic stroke, atrial fibrillation, valvular heart disease (VHD), chronic obstructive pulmonary disease (COPD), estimated glomerular filtration rate (eGFR), hemoglobin, total cholesterol level, low-density lipoprotein cholesterol level, high-density lipoprotein cholesterol level, triglyceride level, and alcohol use disorder (AUD). Additional covariate definitions are provided in eTable 1 in Supplement 1.

### Statistical Analysis

Descriptive analyses compared baseline characteristics by depression status using Wilcoxon rank sum test for continuous variables and χ^2^ test for categorical variables. Kaplan-Meier curves for time to HF event were stratified by depression status, sex, and self-identified race and ethnicity. Unadjusted and age-adjusted incidence rates of depression and HF and their 95% CIs were computed using quasi-Poisson regression and reported for the full cohort and each subgroup of interest (depression status, sex, and self-identified race and ethnicity). The covariate-adjusted incidence rate differences were estimated by multiplying the incidence rate among patients without depression by the proportional change indicated by the fully adjusted hazard ratio (HR) and CI. This approximation method calibrates the reference population to people without depression and yields an inference consistent with the Cox proportional hazards regression model that estimated the HR. All incidence rates are reported as number of cases per 10 000 person-years.

The primary outcome—time to incident HF—was censored at a patient’s last medical contact, death, end of study, or new depression diagnosis. Censoring at the new depression diagnosis allowed a clear comparison between patients with depression at baseline and patients without depression at baseline and without a depression diagnosis during follow-up. The primary analysis studied the adjusted association of depression with the primary outcome of HF. The secondary analysis compared patients with depression at baseline to patients without depression at baseline regardless of their depression diagnosis during follow-up. The secondary analysis was more conservative because its comparator group included both patients with and without a depression diagnosis. While the primary analysis focused on prevalent depression and was censored at the diagnosis of incident depression, a sensitivity analysis with a time-dependent exposure (eMethods in Supplement 1) allowed incident depression cases to switch to depression status at the time of their diagnosis. Even though the exact length of depression history was unknown for the prevalent depression cases, we chose a priori to focus on prevalent depression to select cases with longer depression histories. We also performed a sensitivity analysis of unmeasured confounding for the primary analysis using E-values.^18,19^

In a subgroup analysis, we examined if the association between depression and incident HF existed in the absence of known major HF risk factors by creating a lower-risk cohort. Patients meeting any of the following criteria were excluded from this analysis: body mass index (BMI; calculated as weight in kilograms divided by height in meters squared) of 30 or higher, eGFR of 60 mL/min/1.73 m^2^ or lower, diabetes, ischemic stroke, atrial fibrillation, VHD, COPD, current and former smoking status, and AUD. In an exploratory subgroup analysis, we studied the association of sex, self-identified race and ethnicity, and CAD with HF among the subgroup of patients with baseline depression.

For all analyses, we used a Cox proportional hazards regression model to estimate the cause-specific HR for the etiological association between depression and HF.^20,21,22^ Because death is a competing risk, the Cox model should not be (and was not) used to estimate differences in cumulative incidence of HF. Cox proportional hazards regression model assumptions were investigated graphically (eFigure 5 in Supplement 1). The primary, secondary, and sensitivity analyses with a time-dependent exposure were adjusted for all the aforementioned covariates. Continuous variables were modeled using restricted cubic splines with up to 4 knots.^23^ Missing variables were imputed using predictive mean matching and multiple imputation with chained equations, resulting in 10 imputed datasets.^24,25^ For each imputed dataset, we fit the Cox proportional hazards regression model, and the results from these 10 Cox models were combined using the Rubin rule.^26^ The results are reported as HRs (95% CIs) of developing HF in patients with depression vs patients without depression.

Across all analyses, a statistical significance threshold of *P* = .05 and 2-sided tests were used. Analyses were performed from May 2022 to February 2025 using R, version 3.6.2 (R Project for Statistical Computing).^27^

## Results

Among 3 441 286 veterans who met the medical home definition, 2 843 159 (82.6%) were included in the analytic cohort after exclusions due to death or loss of follow-up (482 302), heart transplant (1209), and preexisting or incident HF diagnosis (114 616) within 1 year of meeting the medical home definition (eFigure 1 in Supplement 1). Participants had a median (IQR) age of 54 (49-59) years; included 165 240 females (5.8%) and 2 677 919 males (94.2%); and 556 914 (19.6%) self-identified as Black, 144 485 (5.1%) as Hispanic, 1 975 068 (69.5%) as White, and 99 011 (3.5%) as other race or ethnicity. In general, participants were overweight (median [IQR] BMI, 29 [26-33]) with a high prevalence of cardiometabolic disease, including 68.5% with hypertension (controlled or uncontrolled) and 32.2% with diabetes (Table 1). We identified 8.0% of participants (226 247 of 2 843 159) with prevalent depression at baseline. Participants with depression compared with those without depression demonstrated a higher prevalence of COPD (12.9% vs 7.1%), current smoking status (43.2% vs 34.7%), and AUD (35.4% vs 11.3%). Additionally, those with depression were younger compared with those without depression (median [IQR] age, 52 [48-57] years vs 54 [49-59] years). Females proportionally made up a larger percentage of those with prevalent depression than those without depression (11.4% vs 5.3%). Participants were followed up for incident HF over a median (IQR) duration of 6.9 (3.4-11.0) years.

**Table 1.** Characteristics of the Full Cohort^a^

Characteristic	Patients, No. (%)		
	Without depression (n = 2 616 912)	With depression (n = 226 247)	All (N = 2 843 159)
Age, median (IQR), y	54 (49-59)	52 (48-57)	54 (49-59)
Sex			
Male	2 503 875 (95.7)	200 454 (88.6)	2 677 919 (94.2)
Female	139 447 (5.3)	25 793 (11.4)	165 240 (5.8)
Self-identified race and ethnicity			
Black	512 941 (19.6)	43 973 (19.4)	556 914 (19.6)
Hispanic	132 011 (5.0)	12 474 (5.5)	144 485 (5.1)
White	1 814 318 (69.3)	160 750 (71.1)	1 975 068 (69.5)
Other^b^	90 740 (3.5)	8271 (3.7)	99 011 (3.5)
BMI, median (IQR)	29 (26-33)	29 (26-33)	29 (26-33)
Diabetes	842 715 (32.2)	73 838 (32.6)	916 553 (32.2)
CAD	257 641 (9.8)	26 593 (11.8)	284 234 (10.0)
Hypertension	1 788 165 (68.3)	158 414 (70.0)	1 946 579 (68.5)
Ischemic stroke	49 721 (1.9)	6037 (2.7)	55 758 (2.0)
Atrial fibrillation	35 560 (1.4)	2984 (1.3)	38 544 (1.4)
VHD	23 435 (0.9)	3694 (1.6)	27 129 (1.0)
COPD	185 082 (7.1)	29 123 (12.9)	214 205 (7.5)
eGFR, median (IQR), mL/min/1.73 m^2^	85 (75-99)	86 (75-100)	85 (75-99)
Hemoglobin, median (IQR), g/dL	15 (14-16)	15 (14-16)	15 (14-16)
Total cholesterol level, median (IQR), mg/dL	191 (165-219)	193 (167-223)	192 (165-220)
LDL cholesterol level, median (IQR), mg/dL	116 (92-140)	116 (92-142)	116 (92-141)
HDL cholesterol level, median (IQR), mg/dL	43 (36-52)	42 (35-52)	42 (35-52)
Triglyceride level, median (IQR), mg/dL	133 (90-203)	142 (94-218)	134 (90-204)
Smoking status			
Current	908 501 (34.7)	97 734 (43.2)	1 006 235 (35.4)
Former	502 407 (19.2)	35 661 (15.8)	538 068 (18.9)
Never	620 751 (23.7)	44 037 (19.5)	664 788 (23.4)
AUD	296 578 (11.3)	79 987 (35.4)	376 565 (13.2)

Abbreviations: AUD, alcohol use disorder; BMI, body mass index (calculated as weight in kilograms divided by height in meters squared); CAD, coronary artery disease; COPD, chronic obstructive pulmonary disease; eGFR, estimated glomerular filtration rate; HDL, high-density lipoprotein; LDL, low-density lipoprotein; VHD, valvular heart disease.

To convert hemoglobin level to grams per liter, multiply by 10.0; and LDL, HDL, total cholesterol to millimoles per liter, multiply by 0.0259; and triglycerides to millimoles per liter, multiply by 0.0113.

^a^ Characteristics of patients with vs without depression were compared using Wilcoxon test for continuous variables and Pearson χ^2^ test for categorical variables. All *P* values were <.001. Variables were missing at the following percentages: 7.5% for race and ethnicity, 2.6% for BMI, 22.3% for smoking status, 4.0% for hypertension, 15.0% for LDL, 12.8% for HDL, 12.8% for triglycerides, 9.2% for hemoglobin, and 5.5% for eGFR.

^b^ Other race and ethnicity includes American Indian or Alaska Native, Asian, Native Hawaiian or Other Pacific Islander, and other (which is undefined in the Veterans Affairs dataset).

**Figure 1.**
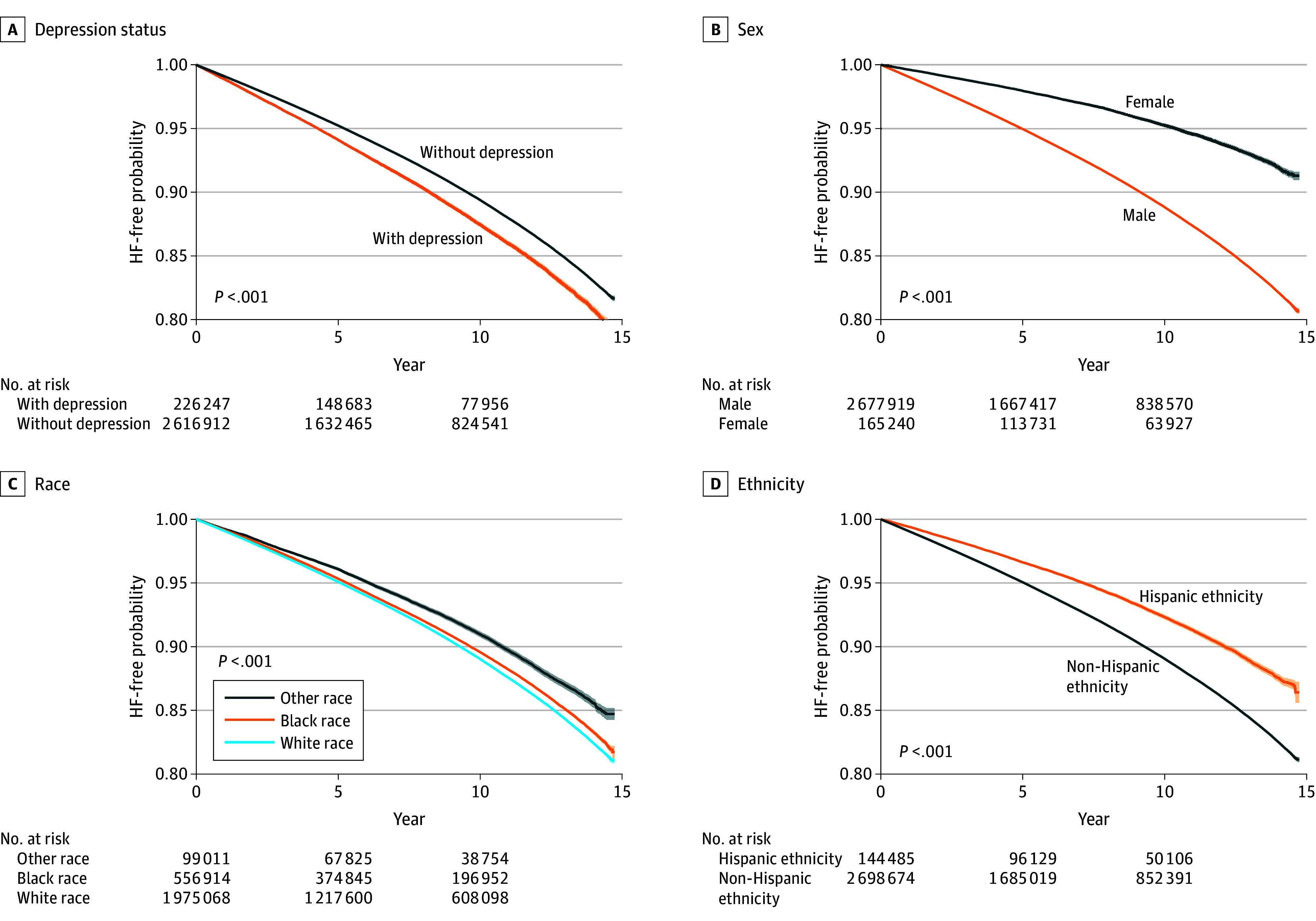
Heart Failure (HF)–Free Survival Stratified by Depression Status, Sex, and Self-Identified Race and Ethnicity Patients were evaluated over a 14-year period for development of incident HF. Other race includes American Indian or Alaska Native, Asian, Native Hawaiian or Other Pacific Islander, and other (which is undefined in the Veterans Affairs dataset). Shaded areas represent 95% CIs.

Participants with depression demonstrated consistently higher incident HF rates compared with those without depression (136.9 [95% CI, 132.2-141.7] cases per 10 000 person-years vs 114.6 [95% CI, 113.4-115.9] cases per 10 000 person-years, respectively) (Table 2). Combined incident HF rates were higher among males compared with females (121.0 [95% CI, 119.7-122.3] cases per 10 000 person-years vs 51.3 [95% CI, 48.4-54.4] cases per 10 000 person-years) and lower in Black vs White individuals (113.5 [95% CI, 110.9- 116.2] cases per 10 000 person-years vs 118.2 [95% CI, 116.8-119.7] cases per 10 000 person-years, respectively). Among patients with depression, Black females had slightly higher rates than White females (76.0 [95% CI, 59.5-97.0] cases per 10 000 person-years vs 66.7 [95% CI, 57.9-76.9] cases per 10 000 person-years, respectively), whereas the rates for Black males were similar to that for White males (142.9 [95% CI, 130.6-156.2] cases per 10 000 person-years vs 146.8 [95% CI, 141.0-152.8] cases per 10 000 person-years, respectively). Rates of HF were lower in females than males regardless of depression status (51.3 [95% CI, 48.4-54.4] cases per 10 000 person-years vs 121.0 [95% CI, 119.7-122.3] cases per 10 000 person-years). In unadjusted time-to-event analyses, we observed a higher probability of HF over time among participants with depression, males, White individuals, and non-Hispanic individuals (Figure 1). In analysis examining HF rates across the age spectrum, we observed higher rates of incident HF with increasing age (eTable 2 in Supplement 1). Depression was associated with higher rates of incident HF at all reported age groups.

**Table 2.** Unadjusted Incident HF Rates by Depression Status for the Full Cohort and Subgroups

Sex and race	HF rates in cases per 10 000 person-years (95% CI)		
	Without depression	With depression	All
Full cohort	114.6 (113.4-115.9)	136.9 (132.2-141.7)	116.5 (115.3-117.7)
No./total No.	214 967/2 616 912	23 239/226 247	238 206/2 843 159
Female	48.1 (45.0-51.4)	68.5 (60.8-77.1)	51.3 (48.4-54.4)
No./total No.	5328/139 447	1411/25 793	6739/165 240
Male	118.8 (117.5-120.2)	146.3 (141.1-151.7)	121.0 (119.7-122.3)
No./total No.	209 639/2 477 465	21 828/200 454	231 467/2 677 919
White	116.4 (114.9-117.9)	137.6 (132.3-143.1)	118.2 (116.8-119.7)
No./total No.	148 937/1 814 318	16 733/160 750	165 670/1 975 068
Black	111.8 (109.2-114.6)	132.9 (122.1-144.6)	113.5 (110.9-116.2)
No./total No.	43 913/512 941	4474/43 973	48 387/556 914
Other race and ethnicity^a^	97.4 (91.2-104.0)	110.9 (94.1-130.6)	98.6 (92.7-104.8)
No./total No.	6986/90 740	750/8271	7736/99 011
Hispanic	80.4 (75.9-85.2)	97.9 (84.2-113.8)	82.0 (77.6-86.6)
No./total No.	8033/132 011	969/12 474	9002/144 485
Non-Hispanic	116.6 (115.2-117.9)	139.3 (134.4-144.4)	118.4 (117.2-119.7)
No./total No.	206 934/2 484 901	22 270/213 773	229 204/2 698 674
Female, White	48.8 (44.9-53.1)	66.7 (57.9-76.9)	51.9 (48.3-55.8)
No./total No.	3257/83 985	927/17 111	4184/101 096
Female, Black	49.5 (43.8-55.9)	76.0 (59.5-97.0)	53.1 (47.5-59.2)
No./total No.	1594/40 104	382/6481	1976/46 585
Female, other race and ethnicity^a^	36.4 (27.3-48.5)	48.5 (31.0-75.9)	38.3 (30.0-49.1)
No./total No.	169/5597	44/1070	213/6667
Male, White	120.1 (118.5-121.7)	146.8 (141.0-152.8)	122.3 (120.7-123.8)
No./total No.	145 680/1 730 333	15 806/143 639	161 486/1 873 972
Male, Black	117.4 (114.5-120.3)	142.9 (130.6-156.2)	119.3 (116.5-122.1)
No./total No.	42 319/472 837	4092/37 492	46 411/510 329
Male, other race and ethnicity^a^	101.6 (95.1-108.6)	120.6 (101.6-143.1)	103.1 (96.9-109.8)
No./total No.	6817/85 143	706/7201	7523/92 344
Low-risk baseline^b^	24.4 (22.7-26.3)	33.1 (25.2-43.4)	24.9 (23.2-26.7)
No./total No.	3236/205 514	255/11 359	3491/216 873

Abbreviation: HF, heart failure.

^a^ Other race and ethnicity includes American Indian or Alaska Native, Asian, Native Hawaiian or Other Pacific Islander, and other (which is undefined in the Veterans Affairs dataset).

^b^ Low-risk baseline subgroup includes patients with a body mass index lower than 30; with an estimated glomerular filtration rate higher than 60 mL/min/1.73 m^2^; without diabetes, ischemic stroke, atrial fibrillation, valvular heart disease, chronic obstructive pulmonary disease, or alcohol use disorder; and without a current or former smoking status.

**Figure 2.**
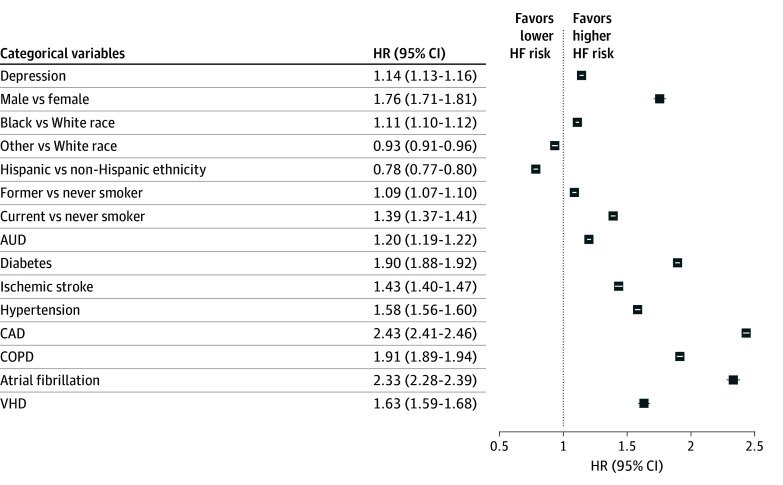
Adjusted Hazard Ratios (HRs) of Incident Heart Failure (HF) for Categorical Variables The HRs and 95% CIs of incident HF are reported for atrial fibrillation, chronic obstructive pulmonary disease (COPD), coronary artery disease (CAD), and valvular heart disease (VHD). AUD indicates alcohol use disorder. Other race includes American Indian or Alaska Native, Asian, Native Hawaiian or Other Pacific Islander, and other (which is undefined in the Veterans Affairs dataset). See eFigure 2 in Supplement 1 for HRs of continuous variables.

The results of the primary analysis are reported as HRs for categorical variables in Figure 2, and HRs for continuous variables are provided in eFigure 2 in Supplement 1. The primary adjusted analysis comparing patients with depression at baseline to patients without depression at and after baseline (patients were censored at new depression diagnosis) showed that depression was associated with a 14.0% increase in the hazard of incident HF (HR, 1.14; 95% CI, 1.13-1.16). A more conservative secondary analysis comparing patients with baseline depression to patients without baseline depression but who may or may not develop depression after baseline (patients were not censored at depression diagnosis) revealed an attenuated association (HR, 1.10; 95% CI, 1.08-1.11). The sensitivity analysis with a time-dependent depression status showed a similar association between depression and incident HF (HR, 1.09; 95% CI, 1.08-1.10).

The E-value sensitivity analysis showed that an unmeasured confounder would need to be associated with HF and with depression with a risk ratio of 1.51 or higher to render the results of the primary analysis inconclusive (ie, not significant). To judge the likelihood of unmeasured confounding to this degree, the E-value should be interpreted within the context of the known potential confounders that have and have not been accounted for in the primary analysis.

**Figure 3.**
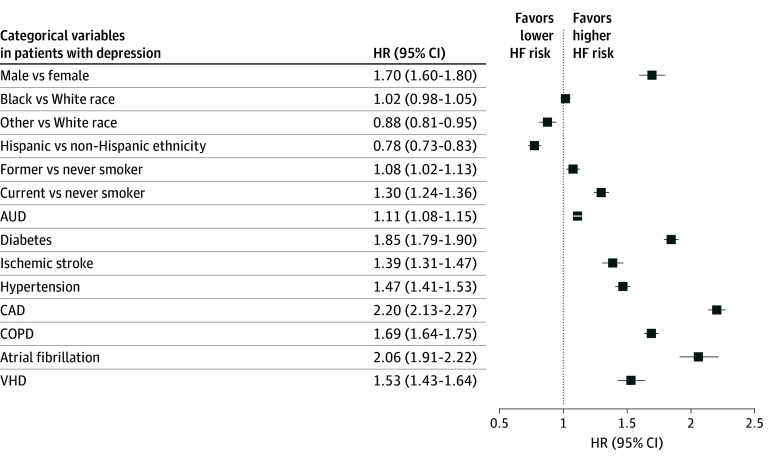
Adjusted Hazard Ratios (HRs) of Incident Heart Failure (HF) for Categorical Variables in the Subcohort of Veterans With Prevalent Depression The HRs and 95% CIs of incident HF are reported for atrial fibrillation, chronic obstructive pulmonary disease (COPD), coronary artery disease (CAD), and valvular heart disease (VHD). AUD indicates alcohol use disorder. Other race includes American Indian or Alaska Native, Asian, Native Hawaiian or Other Pacific Islander, and other (which is undefined in the Veterans Affairs dataset).

In a subanalysis restricted to patients with depression, the hazard for incident HF was higher among males than females (HR, 1.70; 95% CI, 1.60-1.80) and among patients with vs without traditional HF risk factors (eg, HR for patients with vs without CAD was 2.20 [95% CI, 2.13-2.27]). Self-identified Black race was not associated with increased HF risk in the subgroup of patients with depression (HR, 1.02; 95% CI, 0.98-1.05) (Figure 3).

We also examined the association between prevalent depression and incident HF in a low-risk cohort free from comorbidities (eTable 3 in Supplement 1). The analysis of unadjusted incidence rates showed that even among otherwise low-risk individuals, depression was associated with a higher incidence rate of HF compared with individuals without depression (33.1 [95% CI, 25.2-43.4] cases per 10 000 person-years vs 24.4 [95% CI, 22.7-26.3] cases per 10 000 person-years, respectively) (eTable 4 in Supplement 1). Overall rates among subgroups were lower regardless of sex, race and ethnicity, or depression status compared with the full cohort, suggesting that comorbid disease plays a major role in HF. HF rates between Black and White veterans were similar in the analysis restricted to otherwise low-risk individuals (28.2 [95% CI, 24.5-32.5] cases per 10 000 person-years vs 24.4 [95% CI, 22.4-26.5] cases per 10 000 person-years, respectively). The adjusted HR for depression in the low-risk cohort was notably higher than in the full cohort (HR, 1.58; 95% CI, 1.39-1.80), suggesting the proportional outcome of depression may depend on baseline risk (eFigure 3 in Supplement 1; HRs for continuous variables are provided in eFigure 4 in Supplement 1). The adjusted median (IQR) incidence rate differences, which estimate an absolute increase in risk, were similar to those of the primary analysis and the low-risk cohort (16.0 [14.9-18.3] cases per 10 000 person-years and 14.2 [9.5-19.5] cases per 10 000 person-years, respectively).

## Discussion

We defined rates and risk of incident HF among patients with depression in the largest US veteran cohort to date, to our knowledge. The main findings were that depression was associated with a 14.0% increased hazard of incident HF independent of sociodemographic and traditional cardiovascular risk factors and that the increased risk remained similar in analyses with time-dependent depression status. The association remained in the more conservative secondary analysis that did not censor for depression, in the sensitivity analysis with a time-dependent depression status, and in the subgroup of low-risk individuals. Unadjusted rates of incident HF in patients with depression were particularly higher among males than females, Black females compared with White females, and older adults vs younger adults.

This study builds on previous work in this area with several strengths in design. The cohort size was notable, offering sufficient power to evaluate subgroups and seemed to be the largest to date in veterans assessed for comorbid HF and depression. Although the majority of VA patients are White males, our analysis also included 165 240 female veterans, 556 914 Black veterans, and 144 485 Hispanic veterans, with a total cohort size an order of magnitude larger than in prior US studies on this topic.^16^ This study included a large sample of female veterans, a unique and understudied cohort at higher risk for cardiovascular disease than the general population due to higher rates of obesity, depression, and posttraumatic stress in addition to having lower rates of traditional cardiovascular risk factor control compared with male veterans.^28,29,30,31,32^ The cohort size also allowed for evaluation of more confounders than other prior studies examining the relationship between depression and cardiovascular diseases.^3,7^ The use of a medical home definition added confidence that we would capture incident HF diagnoses over time despite the retrospective design.

Furthermore, this study was strengthened by the sensitivity analyses, confirming the findings in a low-risk cohort of patients, when allowing new depression diagnosis in the comparator group, and in patients with a time-dependent depression diagnosis. More specifically, the use of multiple models demonstrated a consistent and robust finding, with each model having its own strengths. The primary analysis had a conservative design, with a fixed baseline and adjustment for covariates (some of which could be considered candidate mediators that may have underestimated the finding), but lacked clarity on the implications of prevalent depression. The sensitivity analysis with a time-dependent depression status offered useful follow-up with inclusion of incident depression. Prior studies examined the implications of comorbid depression and HF for clinical outcomes, whereas this study is novel for its focus on the independent association of depression with incident HF. In addition, the low-risk cohort reduced the potential for confounding related to comorbidities or the mediation of association with adjustment for covariates.

Depression is a leading cause of disability around the world, affecting 4.4% of the world’s population (322 million people), and this rate continues to increase.^33^ Thus, depression remains a widely prevalent disease and a risk factor for HF that may be modifiable. The association between depression and HF is particularly concerning among adolescents and young adults, a population with rapidly increasing rates of depression diagnosis that could substantially impact future HF incidents.^2,10,34^ Because many adolescents and young adults do not have traditional cardiovascular risk factors for HF, such as hypertension, obesity, or systolic dysfunction, a diagnosis of depression may serve as an important indicator in early identification and prevention of HF.^35,36,37,38^ A recent study using the National Health and Nutrition Examination Surveys also suggested increased rates of depression among younger adults (under age 65 years) with HF compared with older adults.^39^

The association between comorbid depression and HF is complex. We attempted to account for known confounders, but unmeasured confounding remains likely, including contributions from sympathetic tone, diet, and sedentary behavior. Chronically elevated sympathetic tone has adverse cardiovascular outcomes, and acute surges may predispose individuals to stress cardiomyopathy, a form of HF. It is also possible that our binary definition of tobacco and alcohol use underestimated the implications of these behaviors for HF risk.^40^

The current American College of Cardiology and the American Heart Association clinical practice guidelines recommend evaluation of symptoms of depression in patients with HF due to risk of poor self-care, rehospitalization, and all-cause mortality.^41,42,43,44^ Our findings support these recommendations for enhanced vigilance in assessment of HF signs and symptoms among patients with depression. Clinical HF guidelines also note the challenge of creating more specific recommendations regarding depression treatment due to limited evidence of effectiveness, including the impact of conventional treatment with antidepressants in prior studies.^3,45,46,47^ We considered assessing the association of depression treatment with HF risk but were concerned by high rates of antidepressant use among patients without a documented depression diagnosis at the medical home date (17.8% [506 521 of 2 843 159]). Therefore, we had low confidence about the indication for antidepressant use. Such widespread use of antidepressants may be attributed to treatment of other mental health disorders or off-label use for indications such as chronic pain. Nonetheless, the association between treatment for depression and incident HF outcomes warrants further evaluation, including trials of pharmacologic and nonpharmacologic interventions, such as cognitive behavioral therapy, which has demonstrated success in patients with HF.^48,49^

### Limitations

This study has several limitations. Older data were used due to the retrospective cohort identified via electronic health records and diagnoses identified by billing codes through 2015. Rigorous data were lacking on treatment for depression or associated socioeconomic risk factors. The focus of this study remained on a heterogenous group of depression diagnoses without making a comparison to other mental health disorders with worse cardiovascular outcomes, such as anxiety and posttraumatic stress disorder. The reliance on billing codes for identifying diagnoses introduced potential for misclassification bias, especially given that symptoms of depression and HF can overlap (eg, fatigue and insomnia). However, this study was less likely confounded by relative clinical care quality because VA facilities, when assessed nationally, demonstrate no substantial differences from non-VA facilities.^50,51,52^

## Conclusions

In this cohort study of US veterans, depression was associated with an increased hazard of incident HF after controlling for traditional sociodemographic and cardiovascular risk factors. Higher rates of incident HF in patients with depression remained similar in the sensitivity analysis with a time-dependent depression status and among a low-risk cohort without other associated comorbidities at baseline. Further study is warranted to ascertain whether earlier recognition and treatment of depression can modify the risk of incident HF and to characterize optimal care for these patients.

## Notes

Supplement 1.
eMethods.
**eTable 1.** Disease Definitions **eTable 2.** Age-Adjusted Incidence Rates of HF by Depression Status **eTable 3.** Cohort Characteristics of the Low-Risk Cohort **eTable 4.** HF Incidence Rates for Low-Risk Cohort by Sub-groups of Sex, Race, and Depression Status **eFigure 1.** Patient Inclusion **eFigure 2.** Adjusted Hazard Ratios of HF for Continuous Variables (Primary Analysis) **eFigure 3.** Adjusted Hazard Ratios of Incident HF for Categorical Variables in the Low-Risk Cohort **eFigure 4.** Adjusted Hazard Ratios of HF for Continuous Variables in the Low-Risk Cohort **eFigure 5.** Depression Beta Coefficient Based on Schoenfeld Residuals
eReferences.

Supplement 2.
Data Sharing Statement
