# Supplementary material for: Depression and Heart Failure in US Veterans
Source: JAMA Netw Open. 2025 May 8;8(5):e259246. doi: 10.1001/jamanetworkopen.2025.9246 (PMC12062911; doi:10.1001/jamanetworkopen.2025.9246)
Supplement: Supplement 1. — eMethods. eTable 1. Disease Definitions eTable 2. Age-Adjusted Incidence Rates of HF by Depression Status eTable 3. Cohort Characteristics of the Low-Risk Cohort eTable 4. HF Incidence Rates for Low-Risk Cohort by Sub-groups of Sex, Race, and Depression Status eFigure 1. Patient Inclusion eFigure 2. Adjusted Hazard Ratios of HF for Continuous Variables (Primary Analysis) eFigure 3. Adjusted Hazard Ratios of Incident HF for Categorical Variables in the Low-Risk Cohort eFigure 4. Adjusted Hazard Ratios of HF for Continuous Variables in the Low-Risk Cohort eFigure 5. Depression Beta Coefficient Based on Schoenfeld Residuals eReferences. [file jamanetwopen-e259246-s001.pdf]

Supplemental Online Content

Pfaff JL, Eden SK, Kundu S, et al. Depression and heart failure in US veterans. *JAMA Netw Open*. 2025;8(5):e259246. doi:10.1001/jamanetworkopen.2025.9246

eMethods.
eTable 1. Disease Definitions
eTable 2. Age-Adjusted Incidence Rates of HF by Depression Status
eTable 3. Cohort Characteristics of the Low-Risk Cohort
eTable 4. HF Incidence Rates for Low-Risk Cohort by Sub-groups of Sex, Race, and Depression Status
eFigure 1. Patient Inclusion
eFigure 2. Adjusted Hazard Ratios of HF for Continuous Variables (Primary Analysis)
eFigure 3. Adjusted Hazard Ratios of Incident HF for Categorical Variables in the Low-Risk Cohort
eFigure 4. Adjusted Hazard Ratios of HF for Continuous Variables in the Low-Risk Cohort
eFigure 5. Depression Beta Coefficient Based on Schoenfeld Residuals
eReferences.

This supplemental material has been provided by the authors to give readers additional information about their work.

© 2025 Pfaff JL et al. *JAMA Network Open*

eMethods.

- Comorbidity definitions:
  Diabetes was identified using a previously validated metric that incorporates serum glucose, hemoglobin A1c, antidiabetic agent use, or at least 1 inpatient or 2 outpatient ICD-9/10 codes for diabetes.[1] BMI was defined as the value closest to medical home (reported or calculated from height and weight) and smoking status was categorized as current, past, never were obtained from the VA Health Factors Dataset.

- Additional explanation of time-dependent analysis:
  "To study the association of depression with incident HF, while accounting for both prevalent depression at baseline and incident depression after baseline, we performed a sensitivity analysis using time-dependent Cox proportional hazards regression.[2] The outcome, time to heart failure, was censored at patient's last medical contact, death, or end of the study. The model was adjusted for the same covariates as the primary model. The time-updated variables included depression, valvular heart disease, coronary artery disease, diabetes, chronic obstructive pulmonary disease, ischemic stroke, atrial fibrillation, and age. The age was updated whenever any of the above comorbidities changed. The remaining variables were not time-updated. Continuous variates and missing values were handled similarly to the primary analysis. Robust sandwich variance estimator was used."

- Cox proportional hazards assumptions:
  An analysis of the Schoenfeld residuals showed the association between depression and HF was stronger between the years 2 to 7 and weaker outside that range. However, the association was always positive, reaching as high as 1.19 around year 4 and as low as 1.06 at year 15 (see **Supplemental Figure 5**). We opted to be conservative and present the hazard ratios over the full 15 years of follow-up, understating the strength of the association that would have been estimated over a shorter follow-up period. Moreover, we discourage over-interpreting the timing of the HR strengths because the times are relative to the date of meeting the medical home definition, not the date of incident depression diagnosis or start of depression. Had we used a different definition for medical home, we could have observed a different time period for the peak strength of the HR; however, we would have still observed a positive association.

© 2025 Pfaff JL et al. *JAMA Network Open*

eTable 1: Disease Definitions

DISEASE	DEFINITION (INCLUDING ICD 9 & 10 CODES)
DEPRESSION	296.20-26, 296.30-36, F32.0-5, F32.9, F33.0-1, F33.3-4, F33.41-42, F33.9
HEART FAILURE	425.x, 428.x, I42.x, I50
CORONARY ARTERY DISEASE	Prevalent: 410.x, 411.x, 412.x, 413.x, 414.x, I21.x , I22.x, I252<br>Incident: 410.x, 411.x , I21.x , I22.x (all inpatient only)
PULMONARY EMBOLISM	415.2, 453.2, 453.4, 453.8, 453.9, 453.40, 453.41, 453.43, V125.1, I26.01, I26.02, I26.09, I26.90, I26.92, I26.99, I27.82, T79.0XXA, T79.1XXA, T80.0XXA
DIABETES MELLITUS	1. Glucose (outpatient only) > 200 mg/dl on two separate occasions (any duration apart)<br>2. ICD-9 codes (two outpatient OR one inpatient) PLUS outpatient treatment with an oral hypoglycemic or insulin for > 30 days<br>3. ICD-9 codes (two outpatient OR one inpatient) PLUS glucose (outpatient) > 126 mg/dl on two separate occasions<br>4. Glucose (outpatient) > 200 mg/dl on one occasion PLUS outpatient treatment with an oral hypoglycemic or insulin for > 30 days<br>5. Hemoglobin A1c(inpatient or outpatient) >= 6.5% on two separate occasions (any duration apart)<br>6. Hemoglobin A1c(inpatient or outpatient) >= 6.5% on one occasion PLUS outpatient treatment with an oral hypoglycemics or insulin for > 30 days
ATRIAL FIBRILLATION	427.31, I48.0, I48.1, I48.2, I48.3, I48.4, I48.9
CHRONIC OBSTRUCTIVE PULMONARY DISEASE	490.x, 491.2x, 491.8, 491.9, 496, 492.8, 492.0, J40, J41, J42, J43, J44, E84, J40.x, J41.x, J42.x, J43.x, J44.x, E84.x
ISCHEMIC STROKE	433.01, 433.11, 433.21, 433.31, 433.81, 433.91, 434.00, 434.01, 434.11, 434.91, 436, I63.02, I63.12, I63.22, I63.031-033, I63.039, I63.131-133, I63.139, I63.23x, I63.239, I63.011-.013, I63.019, I63.111-.113, I63.119, I63.211-.213, I63.219, I63.09, I63.19, I63.00, I63.10, I63.20, I63.29, I66.01-.03, I66.09, I66.11-.13, I66.19, I66.21-.23, I66.29, I66.3-.313, I63.319, I63.321-.323, I63.329, I63.331-.333, I63.339, I63.341, I63.342, I63.343, I63.349, I63.39, I63.6, I63.40, I63.411-.413, I63.419, I63.421-.423, I63.429, I63.431-.433, I63.439, I63.441, I63.442, I63.443, I63.449, I63.49, I63.50, I63.511-.513, I63.519, I63.521-.523, I63.529, I63.531-.533, I63.539, I63.541-.543, I63.549, I63.59, I63.8, I63.9, I67.89
HEMORRHAGIC STROKE	430, 431, I60.00, I60.01, I60.02, I60.10, I60.11, I60.12, I60.20-.22, I60.30-.32, I60.4, I60.50-.52, I60.6-.9, I61.0-.6, I61.8-.9
ANEMIA	Hemoglobin <12 g/dL
HYPERTENSION	Categorized as absent (blood pressure < 140/90 mm Hg and no antihypertensive medication), controlled (blood pressure < 140/90, on antihypertensive medication), or uncontrolled (blood pressure ≥140/90 mm Hg regardless of antihypertensive medication use)
DYSLIPIDEMIA	LDL >/= 160 OR HDL < 40 OR Triglycerides >/= 150
CHRONIC RENAL DISEASE	eGFR <30
TOBACCO USE/SMOKING	Categorized as active use per clinical reporting (current), prior but no active use (former), and never use.
ALCOHOL USE DISORDER	291.x, 303.00-03, 303.90-93, 305.00-03, 357.5, 425.5, 535.3, 571.0, 571.1-3, V11.3, F10.x

x = any digit

© 2025 Pfaff JL et al. *JAMA Network Open*

eTable 2: Age-adjusted Incidence rates of HF by Depression Status

	Total unadjusted rates	45 Years	50 Years	55 Years	60 Years	65 Years	70 Years
NO DEPRESSION	114.6 (113.4, 115.9) N= 214,967/2,616,912	75.3 (73.9, 76.7)	98.2 (97.0, 99.5)	128.1 (126.8, 129.5)	167.2 (164.7, 169.7)	218.2 (213.4, 223.0)	284.7 (276.3, 293.3)
DEPRESSION	136.9 (132.2, 141.7) N= 23,239/226,247	96.9 (91.8, 102.2)	126.1 (121.6, 130.7)	164.1 (158.3, 170.1)	213.5 (202.4, 225.3)	277.9 (257.1, 300.3)	361.7 (326.0, 401.2)

Age-adjusted incidence rates of HF (per 10,000 person-years) and their 95% confidence intervals were assessed in age adjusted analysis and reported for ages from 45 to 70 years at 5-year intervals. Results stratified by depression status.

© 2025 Pfaff JL et al. *JAMA Network Open*

eTable 3: Cohort Characteristics of the Low-Risk Cohort

	NO DEPRESSION N=205,514	DEPRESSION N=11,359	COMBINED N = 216,873
**AGE**	55 (49, 60)	53 (47, 58)	54 (49, 60)
**SEX**			
**MALE**	0.90 (205,1514)	0.77 (8,802)	0.89 (193,693)
**FEMALE**	0.10 (20,623)	0.11 (2,557)	0.11 (23,180)
**SELF-IDENTIFIED RACE**			
**WHITE**	0.67 (137,239)	0.69 (7,850)	0.67 (145,089)
**BLACK**	0.22 (44,416)	0.21 (2,395)	0.22 (46,811)
**OTHER**	0.04 (8,208)	0.04 (478)	0.04 (8,686)
**SELF-IDENTIFIED ETHNICITY**			
**HISPANIC**	0.07 (14,241)	0.09 (1,006)	0.07 (15,247)
**BMI**	27 (25,28)	27 (25,28)	27 (25,28)
**DIABETES**	0 (0)	0 (0)	0 (0)
**CAD**	0 (0)	0 (0)	0 (0)
**HYPERTENSION**	0.52 (107,271)	0.55 (6,282)	0.52 (113,552)
**ISCHEMIC STROKE**	0 (0)	0 (0)	0 (0)
**A. FIB**	0 (0)	0 (0)	0 (0)
**VHD**	0 (0)	0 (0)	0 (0)
**COPD**	0 (0)	0 (0)	0 (0)
**EGFR**	84 (75, 96)	84 (75, 97)	84 (75, 96)
**HEMOGLOBIN**	15 (14, 16)	15 (14, 15)	15 (14, 16)
**CHOLESTEROL**	195 (172, 220)	197 (172, 224)	195 (172, 221)
**LDL**	121 (100, 143)	120 (99, 144)	121 (100, 143)
**HDL**	47 (39, 57)	47 (39, 58)	47 (39, 57)
**TRIGLYCERIDES**	108 (74, 161)	114 (77, 175)	108 (74, 162)
**SMOKING**			
**CURRENT**	0 (0)	0 (0)	0 (0)
**FORMER**	0 (0)	0 (0)	0 (0)
**NEVER**	1 (205,514)	1.0 (11,359)	1 (216,873)
**ALCOHOL USE DO**	0 (0)	0 (0)	0 (0)

Categorical values reported as proportions and counts. Continuous values were reported as medians and interquartile ranges. Patient characteristics among depressed and not depressed were compared using Wilcoxon test for continuous and Pearson Chi-square test for categorical variables. All p-values were <0.001 except for Race (0.039), eGFR (0.840), LDL (0.380), and HDL (0.020). Race= self-identified race. CAD = coronary artery disease. A. FIB = atrial fibrillation. VHD = valvular heart disease. COPD = chronic obstructive pulmonary disease. Cholesterol = total cholesterol. Alcohol use DO = alcohol use disorder flag. Variables were missing at the following percentages: Race (7.5%), Hypertension (0.8%), LDL (7.7%), HDL (6.3%), Triglycerides (6.8%), Hemoglobin (3.9%).

© 2025 Pfaff JL et al. *JAMA Network Open*

eTable 4: HF Incidence Rates for Low-Risk Cohort by Sub-groups of Sex, Race, and Depression Status

SUBGROUP	EVENTS	RATES/10,000 PY
**ALL**	3,491/216,873	24.9 (23.2, 26.7)
**MALE**	3,287/193,693	26.4 (24.5, 28.4)
**FEMALE**	204/23,180	13.0 (10.1, 16.9)
**WHITE**	2,255/145,089	24.4 (22.4, 26.5)
**BLACK**	874/46,811	28.2 (24.5, 32.5)
**OTHER**	119/8,686	20.0 (13.3, 30.0)
**HISPANIC**	194/15,247	19.1 (15.3, 23.8)
**NON-HISPANIC**	3,297/201,626	25.4 (23.6, 27.3)
**NO DEPRESSION**	3,236/205,514	24.4 (22.7, 26.3)
**DEPRESSION**	255/11,359	33.1 (25.2, 43.4)

Rates reported by 10,000 person-years (PY) with interquartile range.

© 2025 Pfaff JL et al. *JAMA Network Open*

eFigure 1: Patient Inclusion

Among 3.4 million veterans meeting our medical home definition, 2,843,159 met all inclusion criteria. We excluded 598,127 patients who died, had heart transplant, pre-existing HF or HF diagnosis within one year from meeting medical home definition.

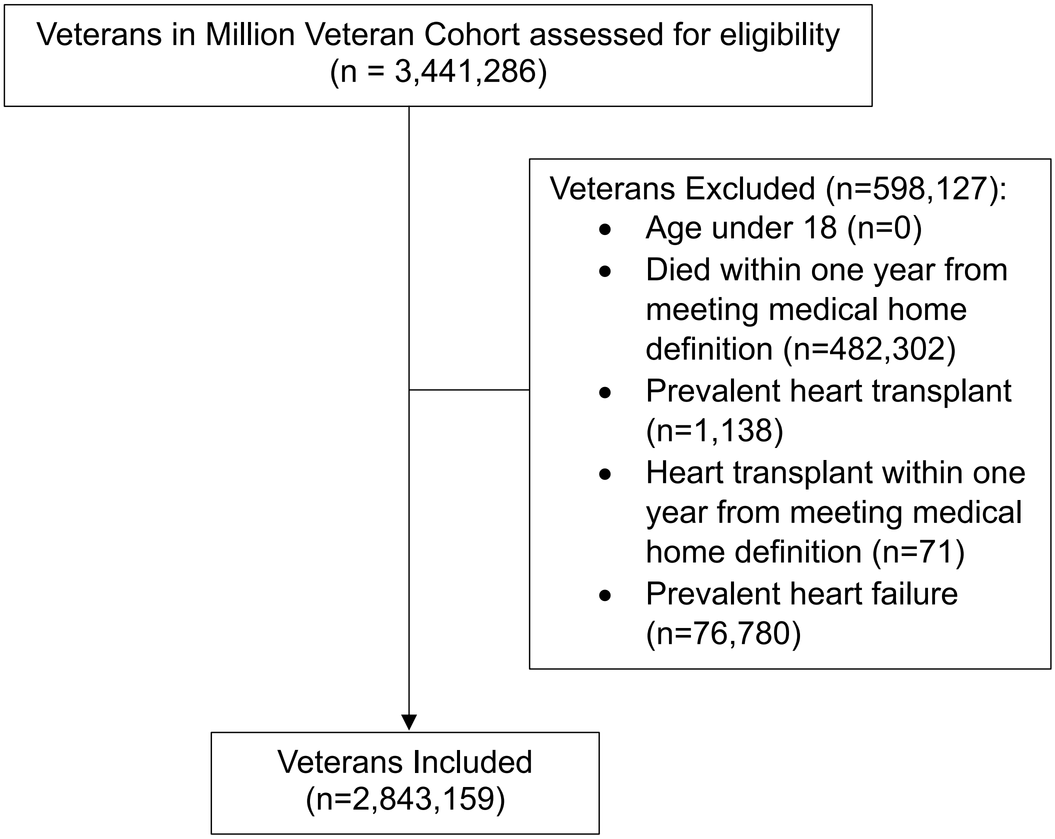

© 2025 Pfaff JL et al. *JAMA Network Open*

eFigure 2: Adjusted Hazard Ratios of HF for Continuous Variables (Primary Analysis)

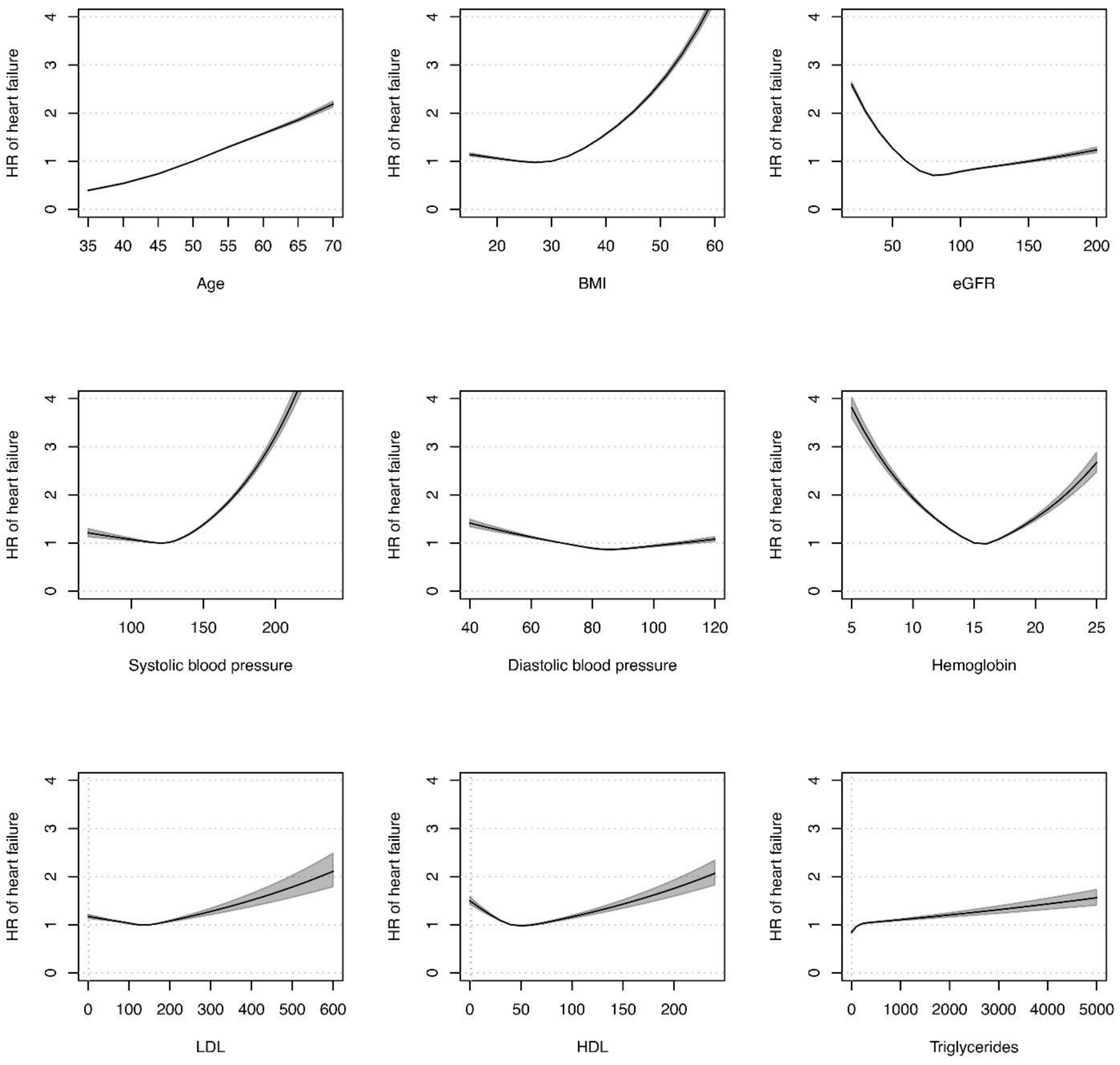

The adjusted hazard ratios and 95% CI of incident HF for continuous covariates are reported as functions over the range of the corresponding variables. The following reference values were used: age (50), BMI (30), eGFR (60), systolic (120) and diastolic (70) blood pressure, hemoglobin (15), LDL (150), HLD (40), triglycerides (150). All of the covariates had statistically significant nonlinear associations with incident HF, all p<0.0001.

© 2025 Pfaff JL et al. *JAMA Network Open*

eFigure 3: Adjusted Hazard Ratios of Incident HF for Categorical Variables in the Low-Risk Cohort

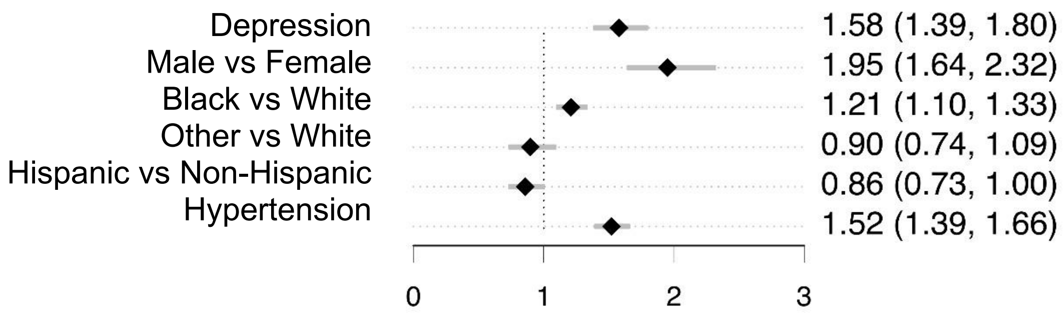

The adjusted hazard ratios and 95% CI of incident HF for categorical variables included in the model; model excludes variables with no conditions present, i.e. DM, Ischemic Stroke, CAD, COPD, AFIB, VHD, former or current smoking, and alcohol use disorder. See Supplemental Figure 4 for hazard ratios of continuous variables.

© 2025 Pfaff JL et al. *JAMA Network Open*

eFigure 4: Adjusted Hazard Ratios of HF for Continuous Variables in the Low-Risk Cohort

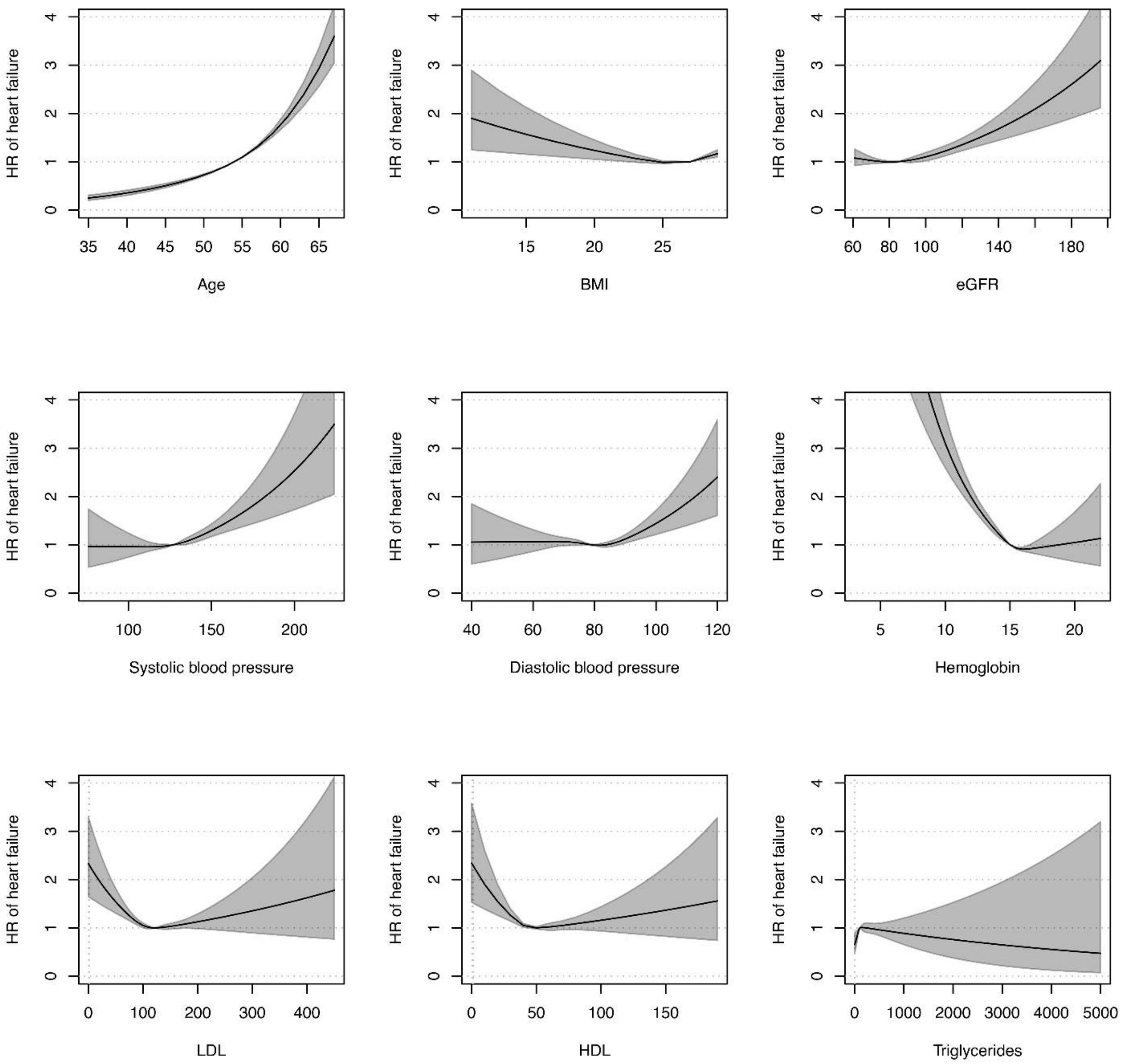

The adjusted hazard ratios and 95% CI of incident HF for continuous covariates are reported as functions over the range of the corresponding variables. The following reference values were used: age (50), BMI (30), eGFR (60), systolic (120) and diastolic (70) blood pressure, hemoglobin (15), LDL (150), HLD (40), and triglycerides (150). Statistically significant nonlinear associations with incident HF were observed for the rest of the covariates (p<0.01) except age (p = 0.008), systolic blood pressure (p=0.038), and triglycerides (p=0.007).

© 2025 Pfaff JL et al. *JAMA Network Open*

eFigure 5: Depression beta coefficient based on Schoenfeld residuals

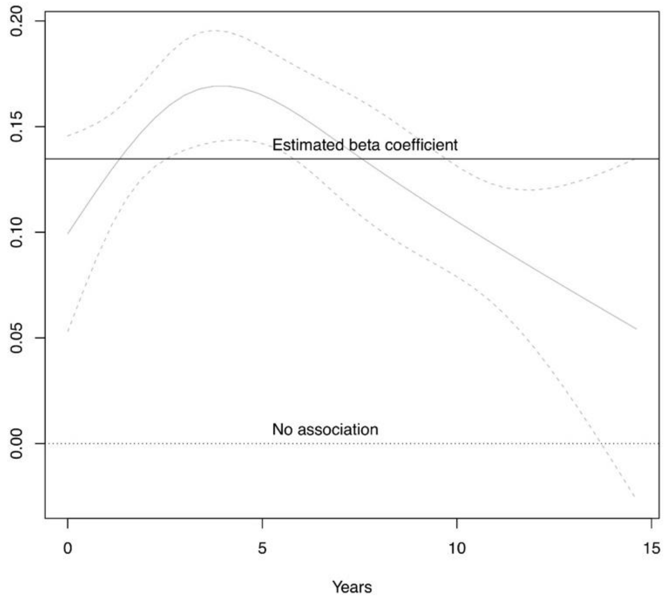

An analysis of the Schoenfeld residuals with x axis as time in years and y axis as the log hazard ratio. This analysis demonstrates the association between depression and HF stronger between the years 2 to 7 and weaker outside that range. However, the association was always positive, reaching as high as 1.19 around year 4 and as low as 1.06 at year 15.

© 2025 Pfaff JL et al. *JAMA Network Open*

eReferences.

1. Duncan MS, Alcorn CW, Freiberg MS, et al. Association between HIV and incident pulmonary hypertension in US Veterans: a retrospective cohort study. Lancet Healthy Longev. Jul 2021;2(7):e417-e425. doi:10.1016/s2666-7568(21)00116-1.

2. Kalbfleisch J, Prentice R. The Statistical Analysis of Failure Time Data John Wiley & Sons, Inc.; 2002.

© 2025 Pfaff JL et al. *JAMA Network Open*
