# Supplementary material for: Depression and Heart Failure in US Veterans
Source: JAMA Netw Open. 2025 May 8;8(5):e259246. doi: 10.1001/jamanetworkopen.2025.9246 (PMC12062911; doi:10.1001/jamanetworkopen.2025.9246)
Supplement: Supplement 2. — Data Sharing Statement [file jamanetwopen-e259246-s002.pdf]

Data Sharing Statement

Pfaff. Depression and Heart Failure in US Veterans. *JAMA Netw Open*. Published May 08, 2025. doi:10.1001/jamanetworkopen.2025.9246

Data
Data available: No

Additional Information
Explanation for why data not available: Because of the identifiable nature of the data, which was collected on US veterans, the authors are not at liberty to share these data with outside investigators. Specific inquiries will be considered on a case-by-case basis upon consultation with the corresponding author.
